# Low medical trust predicts delays in seeking care among U.S. individuals who smoke: Findings from the 2022 HINTS

**DOI:** 10.1016/j.pmedr.2026.103420

**Published:** 2026-02-17

**Authors:** Whitney M. Brymwitt, Timothy J. Williamson

**Affiliations:** Department of Psychological Science, Loyola Marymount University, 1 LMU Drive, Suite 4700, Los Angeles, CA 90045, United States

**Keywords:** Trust, Delayed care seeking, Smoking, Preventive health services, Health behavior

## Abstract

**Objectives:**

Individuals who smoke face increased risk for chronic disease but often delay seeking care, hindering prevention efforts. Low medical trust is a barrier associated with care delays in the general population; less is known about this relationship among people who smoke.

We examined whether low medical trust predicted delays in seeking care among U.S. individuals who smoke (*n* = 431, weighted *n* = 20.8 million), using the 2022 Health Information National Trends Survey.

**Methods:**

We conducted a pre-registered, multivariable logistic regression with medical trust predicting delayed care (within the prior 12 months) as the outcome. Covariates included race, sex, age, education, financial strain, chronic conditions, self-reported health, and insurance.

**Results:**

Lower trust was associated significantly with a higher likelihood of delaying needed medical care (OR = 0.54, 95% CI [0.31, 0.93]). Participants who were Black/African American (vs. non-Hispanic White), aged 65–74 (vs. 18–34), and those who reported better general health were significantly less likely to delay care (all *p* < .05).

**Conclusions:**

Lower trust predicted delayed care-seeking among people who smoke. Future research should explore whether anticipated stigma explains this relationship. Findings highlight the need to bolster trust in healthcare (e.g., communication interventions) to facilitate engagement in preventive care (e.g., smoking cessation, lung cancer screening).

## 1 Introduction

Smoking increases risk for cardiovascular disease, diabetes, and various cancers (e.g., lung, bladder, esophageal) (Caliri et al., 2021). Morbidity and mortality rates for these conditions are high (Yang et al., 2020), and the burdens of tobacco use and tobacco-related disease are substantial (Makate et al., 2020). Preventive care (e.g., routine tobacco assessment), early detection (e.g., lung cancer screening), and risk reduction (e.g., tobacco cessation) can reduce tobacco-related morbidity and mortality (Cho et al., 2024). However, uptake is low among people who smoke (Khanna et al., 2021). People who smoke are more likely to delay seeking care for preventive exams and distressing symptoms (Smith et al., 2016), limiting the impact of these efforts. People who smoke may be reluctant to engage with healthcare due to concerns about stigma, which can undermine trust and limit the effectiveness of clinical communication (Ostroff et al., 2022). Delays are associated with worse outcomes in lung cancer and chronic obstructive pulmonary disease (Locke et al., 2022; Zhang et al., 2022), emphasizing the need to characterize factors that explain care delays among people who smoke.

One possible factor is low trust in the healthcare system (i.e., lack of confidence that medical care will be delivered benevolently, truthfully, and/or competently) (Shukla et al., 2025). People who smoke report high anticipated stigma in healthcare settings (e.g., concerns about being judged or blamed for health problems) and experiences of stigma from clinicians (Madawala et al., 2023; Ostroff et al., 2022), which are antecedents to lower medical trust (Shukla et al., 2025). Stigma can undermine trust in clinical encounters and hinder supportive patient-clinician communication (Ostroff et al., 2022), whereas high medical trust can facilitate positive patient-clinician communication and promote care engagement (Hillen et al., 2011). Low medical trust is associated with delayed care seeking in the general population (LaVeist et al., 2009) and with lower lung cancer screening participation (Carter-Harris et al., 2020), but less is known about whether lower medical trust among those who smoke predicts delays in seeking care. In this study, we used data from a nationally representative sample of U.S. adults to test the hypothesis that among individuals who smoke, lower trust in the healthcare system would predict a higher likelihood of delaying needed medical care. Additionally, we tested whether lower medical trust would predict lower self-reported healthcare utilization.

## 2 Methods

### 2.1 Study design and population

A sample of *N* = 6140 participants was recruited by the National Cancer Institute to complete the 2022 Health Information National Trends Survey (HINTS) Cycle 6 between March 7th and November 8th, 2022. This sampling method produced a nationally representative sample that can be generalized to the U.S. population (HINTS 6 Methodology Report, 2023). This study was reviewed and designated as exempt research under 45 CFR 46.104 by the Westat IRB (Project #6632.03.51, approved 5/10/2021) and the Loyola Marymount University IRB (Project #2025FA54-R, approved 4/10/2025). Informed consent was obtained from all participants.

We included participants who reported current smoking and no history of cancer (to enhance relevance for preventive care). We excluded participants who did not need medical care in the prior 12 months (*n* = 38) or had incomplete data (*n* = 77), resulting in an analyzed sample of *n* = 431 (weighted population estimate: 20,833,472).

### 2.2 Measures

Medical trust was assessed with: “*How much do you trust the healthcare system (for example, hospitals, pharmacies, and other organizations involved in healthcare)?*” on a 4-point Likert scale ranging from 1 (a lot) to 4 (not at all). Items were reverse-scored; higher scores indicate higher trust.

Delay in seeking care was assessed with: *“In the past 12 months, did you delay or not get medical care you felt you needed - such as seeing a doctor, a specialist, or other health professional?”* Participants could select “yes”, “no, I received the medical care I felt I needed”, or “I did not need any medical care in the past 12 months.” Responses of “yes” were coded as 1, “no, I received the medical care I felt I needed” were coded as 0; those who reported “I did not need any medical care in the past 12 months” were excluded from analysis.

Self-rated general health was assessed with, *“In general, would you say your health is…,”* with responses scored from 1 (“Poor”) to 5 (“Excellent”), such that higher scores indicate better self-rated health. Comorbid chronic conditions was assessed by computing a new variable (range 0–4) that summed participants' self-reported “yes” responses to four questions asking whether a doctor or other health professional had ever diagnosed them with: diabetes or high blood sugar; high blood pressure or hypertension; a heart condition (e.g., heart attack, angina, congestive heart failure); or a chronic lung condition (e.g., asthma, emphysema, chronic bronchitis).

Healthcare utilization was assessed with the question: “*In the past 12 months, not counting times you went to an emergency room, how many times did you go to a doctor, nurse, or other health professional to get care for yourself?”*, with responses recoded so that “none” was coded as 0 and 1+ visits coded as “1.”

Sociodemographic information included race (Non-Hispanic White, Non-Hispanic Black or African American, Hispanic, Non-Hispanic Asian, Non-Hispanic Other), sex at birth (male, female), age (18–34, 35–49, 50–64, 65–74, 75+ years), education (less than high school, high school, some college, college graduate or higher), financial strain (Likert scale from 1 “Living comfortably on present income” to 4 “Finding it very difficult on present income” with higher scores indicating higher strain), and insurance (no, yes).

### 2.3 Statistical analysis

Trust in the healthcare system was included as a predictor in two multivariable models: a logistic regression predicting delays in seeking medical care (yes/no) and a linear regression predicting healthcare utilization. Race, sex at birth, age, education, financial strain, chronic conditions, self-rated general health, and insurance were entered as covariates. The variance inflation factor was low (VIF = 1.27), indicating no multicollinearity concerns. Analyses used jackknife replication weights. We constructed 95% confidence intervals (CIs) for the estimated odds ratios (ORs), which were used to determine statistical significance. This study was preregistered on Open Science Framework (https://osf.io/v6hcp/?view_only=8c8aef5696e54222a43bc88741d0d923), following best practices for pre-existing data. The data is publicly available (https://hints.cancer.gov/data/). All statistical analyses were conducted using STATA/SE 18.0.

## 3 Results

In the analyzed sample (*n* = 431, representing 20.8 million individuals), weighted proportions indicated that *n* = 252 (51.76%) were female; *n* = 250 (68.21%) were non-Hispanic White, *n* = 93 (16.01%) Black or African American, *n* = 56 (7.91%) Hispanic, *n* = 13 (3.35%) non-Hispanic Asian, (*n* = 19) 4.52% non-Hispanic “Other”; and an average (weighted) age of 47.91 (*SE* = 1.38). Weighted estimates indicated that *n* = 20 (2.7%) reported no trust in the healthcare system, *n* = 73 (19.39%) indicated they had little trust, *n* = 201 (42.40%) indicated they were somewhat trustful, and *n* = 137 (35.53%) indicated that they were very trustful. Nearly half (*n* = 192; 46.03%) reported delaying needed medical care in the past year.

As hypothesized, for each unit increase in trust, the odds of delaying needed healthcare were 46% lower (OR = 0.54, SE = 0.15, 95% CI [0.31, 0.93]), controlling for covariates (Table 1). Additionally, Black participants were less likely than non-Hispanic White participants to delay seeking care (OR = 0.30, SE = 0.16, 95% CI [0.10, 0.88]). Participants aged 65–74 were less likely to delay seeking care, compared to participants aged 18–34 (OR = 0.07, SE = 0.09, 95% CI [0.01, 0.76]). Finally, higher self-reported health predicted a lower likelihood of delaying medical care (OR = 0.45, SE = 0.14, 95% CI [0.25, 0.83]. Results from a parallel unweighted logistic regression (using the same variables and sample), indicated that 27% of the variance in delay was explained by the model (McFadden pseudo-R^2^ = 0.27); however, caution is warranted in interpretation, as all substantive inference is based on weighted analyses.

**Table 1:** Logistic Regression Predicting Delays in Seeking Needed Medical Care among U.S. Adults who Report Current Smoking in 2022 (n = 431, estimated population size 20,833,472).

Variable	Adjusted odds ratio	95% confidence interval	Unweighted proportions of sample reporting delays in needed medical care by variable category	Weighted population estimates of reporting delays in needed medical care by variable category
Age				
18–34 (referent)	1		31/48 (64.6%)	2,472,132/3,888,730 (63.6%)
35–49	0.22	[0.03,1.33]	61/122(50.0%)	2,986,832/6,997,079 (42.7%)
50–64	0.30	[0.04,2.38]	76/169 (45.0%)	3,656,313/7,946,681 (46.0%)
65–74	0.07*	[0.01,0.76]	22/74 (29.8%)	412,777/1,585,818 (26.0%)
75+	0.04	[0.00,1.48]	2/18 (11.1%)	61,270/415,165(14.8%)
Sex				
Male (referent)	1		80/179 (44.7%)	4,848,811/10,050,218 (48.2%)
Female	0.53	[0.18,1.51]	112/252 (44.4%)	4,740,513/10,783,254 (44.0%)
Education				
Less than High School (referent)	1		22/46 (47.8%)	1,084,313/1,768,439 (61.3%)
High School Graduate	0.19	[0.02,1.98]	42/107 (39.3%)	1,672,776/5,262,867 (31.8%)
Some College	0.54	[0.05,6.19]	79/168 (47.0%)	5,444,436/10,886,849 (50.0%)
College Graduate or More	0.42	[0.03,5.06]	49/110 (44.5%)	1,387,799/2,915,318 (47.6%)
Race and ethnicity				
Non-Hispanic White (referent)	1		112/250 (44.8%)	6,674,864/14,211,325 (47.0%)
Non-Hispanic Black or African American	0.30*	[0.10,0.88]	38/93 (40.9%)	893,950 /3,335,856 (26.8%)
Hispanic	0.56	[0.14,2.23]	23/56 (41.1%)	797,294/1,647,686 (48.4%)
Non-Hispanic Asian	2.99	[0.11,78.10]	7/13 (53.8%)	550,326/696,946 (79.0%)
Non-Hispanic Other	4.34	[1.00,18.89]	12/19 (63.2%)	672,890/941,660 (71.5%)
Insurance Coverage				
Yes (referent)	1		169/387 (43.7%)	8,745,603/18,933,657 (46.2%)
No	0.84	[0.25, 2.77]	23/44 (52.3%)	843,721/1,899,815 (44.4%)
				
			Unweighted Mean *(SD)*	Weighted Mean *(SE)*
Trust in Healthcare System				
	0.54*	[0.31,0.93]	3.06 *(0.82)*	3.11 *(0.08)*
Financial Strain				
	1.37	[0.91, 2.06]	2.34 *(0.97)*	2.09 *(0.07)*
Self-Rated General Health				
	0.45*	[0.25, 0.83]	2.91 *(0.92)*	3.03 *(0.09)*
Number of Comorbid Chronic Conditions				
	0.78	[0.52, 1.18]	1.07 *(1.08)*	0.87 *(0.08)*

Note: SD = Standard Deviation; SE = Standard Error; * = *p* < .05.

For each unit increase in trust, the odds of visiting a healthcare provider were 2.28 times higher (OR = 2.28, SE = 0.75, 95% CI [1.17, 4.43]), controlling for covariates. Results from a parallel unweighted logistic regression indicated that 29% of the variance in delay was explained by the model (McFadden pseudo-R^2^ = 0.29). There was no significant difference in healthcare visit occurrence between participants who reported delaying care and those who did not (F(1,49) = 1.01, *p* = .32).

## 4 Discussion

**Fig. 1:**
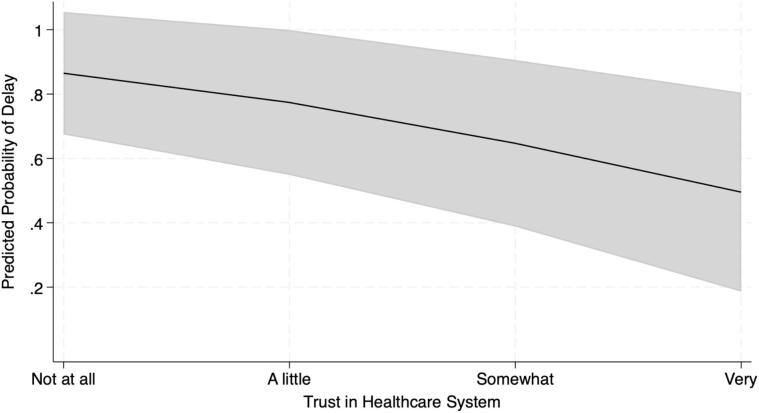
Predicted Probability of Reporting Delay in Needed Medical Care by Trust in the Healthcare System among U.S. Adults who Smoke (n = 431, estimated population size 20,833,472). Note: Lower trust in the healthcare system (M = 3.12, SE = 0.08) was independently associated with higher probability of delays in seeking needed medical care (*n* = 192 respondents [46.03%] reported a delay in seeking care), adjusting for age, sex, education, race and ethnicity, financial strain, self-rated health, comorbid chronic conditions, and insurance coverage. Shaded area indicates 95% confidence interval for the line of best fit from the multivariable logistic regression model.

In this nationally representative sample, lower trust in the healthcare system was associated with higher odds of delaying needed medical care among people who smoke (Fig. 1). These findings are consistent with studies in the general population (LaVeist et al., 2009), suggesting that trust in the healthcare system has a similar relationship to healthcare usage among those who smoke. Prior research has linked smoking-related stigma to low trust, so it is possible that the observed relationship between lower trust and delay in care reflects anticipated stigma about smoking. However, because the current study did not assess stigma, future research should explore stigma as a mediator.

Unexpectedly, Black participants were less likely to report a delay in seeking care than White participants. This is notable because Black Americans are more likely to be diagnosed with smoking-related illnesses (e.g., lung cancer) at later stages (Kratzer et al., 2024). These findings align with research demonstrating that Black Americans are more likely to adopt early lung cancer screening, despite disparities in incidence and mortality (Shusted et al., 2024). Future research should explore other factors (e.g., delayed referrals, structural racism) that may contribute to these disparities. Adults aged 65–74 were less likely to delay care, compared to those aged 18–34. Those between 65 and 74 are more likely to have preexisting conditions and may engage more regularly with care (NHIS Adult Summary Health Statistics, 2025). More frequent appointments may provide more opportunities to raise health concerns. Finally, participants with higher self-rated health were less likely to delay care than those with poorer self-rated health. This pattern is concerning because it suggests that individuals who may have greater healthcare needs are disproportionately delaying care.

Lower trust in the healthcare system was associated not only with a higher likelihood of delaying care but also with lower self-reported healthcare utilization, suggesting that medical trust is linked to both the timing and overall engagement with healthcare among individuals who smoke. These findings are consistent with prior research showing that among individuals reporting a persistent cough, those who smoke were less likely to seek medical care (46%) compared to those who did not smoke (55%; Smith et al., 2016).

This study has several limitations. The delay in care measure did not specify the type of care (e.g., preventive vs. symptom-driven), the reason for delay (e.g., stigma, cost, time), or the duration of delay (e.g., postponed vs. cancelled). The cross-sectional design limits causal inference. Trust was measured with one item, and stigma was not assessed. Given the modest variance explained by trust, future studies should explore other factors (e.g., anticipated stigma) that may influence care-seeking behavior. Nevertheless, this study has several strengths. It used data from a large, nationally representative sample, allowing for generalizability. It also addressed a gap in the literature by examining trust in the healthcare system specifically among people who smoke, a population at risk for chronic illness and underuse of healthcare services.

## 5 Conclusion

This study highlights the importance of building trust in the healthcare system among individuals who smoke. Given their elevated risk and greater likelihood of delaying care, efforts to enhance trust could facilitate care engagement. Approaches to enhance trust could include facilitating shared decision making (e.g., lung cancer screening) and clinician trainings that foster empathic communication skills. Healthcare institutions could implement policies to bolster trust, including community advisory boards and engagement strategies such as Bridging Research and Accurate Information through Dialogue (Stephenson-Hunter et al., 2023). Research should explore whether stigma contributes to the link between low trust and delayed care and evaluate the characteristics of individuals who reported not needing care, given that engagement in care is especially important for individuals who smoke. Strengthening trust and reducing delays in care among people who smoke may support efforts to promote earlier intervention and reduce morbidity.

## CRediT authorship contribution statement

**Whitney M. Brymwitt:** Writing – review & editing, Writing – original draft, Visualization, Methodology, Formal analysis, Data curation, Conceptualization. **Timothy J. Williamson:** Writing – review & editing, Writing – original draft, Supervision, Resources, Project administration, Methodology, Formal analysis, Data curation, Conceptualization.

## Declaration of competing interest

The authors declare the following financial interests/personal relationships which may be considered as potential competing interests: Timothy Williamson reports financial support was provided by the National Cancer Institute. Whitney Brymwitt declares that she has no known competing financial interests or personal relationships that could have appeared to influence the work reported in this paper.

## References

[bb0005] Caliri A.W., Tommasi S., Besaratinia A. (2021). Relationships among smoking, oxidative stress, inflammation, macromolecular damage, and cancer. Mutation Res./Rev. Mutation Res..

[bb0010] Carter-Harris L., Slaven J.E., Monahan P.O., Draucker C.B., Vode E., Rawl S.M. (2020). Understanding lung cancer screening behaviour using path analysis. J. Med. Screen..

[bb0015] Cho E.R., Brill I.K., Gram I.T., Brown P.E., Jha P. (2024). Smoking cessation and short- and longer-term mortality. NEJM Evidence.

[bb0020] Hillen M.A., de Haes H.C.J.M., Smets E.M.A. (2011). Cancer patients’ trust in their physician—a review. Psychooncology.

[bb0025] HINTS 6 Methodology Report (2023). Westat. https://hints.cancer.gov/data/methodology-reports.aspx.

[bb0030] Khanna N., Klyushnenkova E., Rao V., Siegel N., Wolfe S. (2021). Electronic referrals to the tobacco Quitline: implementation strategies in a large health system to optimize delivery of tobacco cessation to patients. Transl. Behav. Med..

[bb0035] Kratzer T.B., Bandi P., Freedman N.D., Smith R.A., Travis W.D., Jemal A., Siegel R.L. (2024). Lung cancer statistics, 2023. Cancer.

[bb0040] LaVeist T.A., Isaac L.A., Williams K.P. (2009). Mistrust of health care organizations is associated with underutilization of health services. Health Serv. Res..

[bb0045] Locke E.R., Young J.P., Battaglia C., Simpson T.L., Trivedi R., Simons C., Fortney J.C., Hebert P., Swenson E.R., Edelman J., Fan V.S. (2022). Care-seeking and delay of care during COPD exacerbations. Npj Primary Care Resp. Med..

[bb0050] Madawala S., Enticott J., Sturgiss E., Selamoglu M., Barton C. (2023). The impact of smoking status on anticipated stigma and experience of care among smokers and ex-smokers with chronic illness in general practice. Chronic Illn..

[bb0055] Makate M., Whetton S., Tait R.J., Dey T., Scollo M., Banks E., Norman R., Pidd K., Roche A.M., Allsop S. (2020). Tobacco cost of illness studies: a systematic review. Nicotine Tob. Res..

[bb0060] NHIS Adult summary health statistics (2025). CDC. https://wwwn.cdc.gov/NHISDataQueryTool/SHS_adult/index.html.

[bb0065] Ostroff J.S., Banerjee S.C., Lynch K., Shen M.J., Williamson T.J., Haque N., Riley K., Hamann H.A., Rigney M., Park B. (2022). Reducing stigma triggered by assessing smoking status among patients diagnosed with lung cancer: De-stigmatizing do and don’t lessons learned from qualitative interviews. PEC Innovation.

[bb0070] Shukla M., Schilt-Solberg M., Gibson-Scipio W. (2025). Medical mistrust: a concept analysis. Nurs. Rep..

[bb0075] Shusted C.S., Eberth J.M., Juon H.-S., Barta J.A. (2024). Characteristics associated with early vs. late adoption of lung cancer screening. Prev. Med. Rep..

[bb0080] Smith C.F., Whitaker K.L., Winstanley K., Wardle J. (2016). Smokers are less likely than non-smokers to seek help for a lung cancer ‘alarm’ symptom. Thorax.

[bb0085] Stephenson-Hunter C., Yusuf Y., Larson R., Campanella J., Gutnick D.N. (2023). What matters to us: bridging research and accurate information through dialogue (BRAID) to build community trust and cultivate vaccine confidence. Prev. Med. Rep..

[bb0090] Yang R., Zhou Y., Wang Y., Du C., Wu Y. (2020). Trends in cancer incidence and mortality rates in the United States from 1975 to 2016. Ann. Trans. Med..

[bb0095] Zhang J., IJzerman M.J., Oberoi J., Karnchanachari N., Bergin R.J., Franchini F., Druce P., Wang X., Emery J.D. (2022). Time to diagnosis and treatment of lung cancer: a systematic overview of risk factors, interventions and impact on patient outcomes. Lung Cancer.

